# Optimizing the Soil Calcium:Magnesium Ratio Improves the Mitragynine Yield and Seedling Growth in Kratom (*Mitragyna speciosa*)

**DOI:** 10.3390/plants15071098

**Published:** 2026-04-03

**Authors:** Nisa Leksungnoen, Tushar Andriyas, Yongkriat Ku-Or, Suthaporn Chongdi, Pichaya Pongchaidacha, Chatchai Ngernsaengsaruay, Suwimon Uthairatsamee, Rossarin Tansawat, Kanjananat Boondum

**Affiliations:** 1 Department of Forest Biology, Faculty of Forestry, Kasetsart University, Bangkok 10900, Thailand; ffornsl@ku.ac.th (N.L.); yongkriat1027@gmail.com (Y.K.-O.); suthaporn.b@gmail.com (S.C.); fforsmu@ku.ac.th (S.U.); 2 Department of Environmental Science, Faculty of Science, Chulalongkorn University, Bangkok 10330, Thailand; 3 Department of Food and Pharmaceutical Chemistry, Faculty of Science, Chulalongkorn University, Bangkok 10330, Thailand; txxnpchya@hotmail.com (P.P.); rossarin.t@pharm.chula.ac.th (R.T.); 4 Center of Excellence in Metabolomics for Life Sciences, Chulalongkorn University, Bangkok 10330, Thailand; 5 Department of Botany, Faculty of Science, Kasetsart University, Bangkok 10900, Thailand; fscicnn@ku.ac.th; 6 Phanangtung Botanical Garden, Department of National Parks, Wildlife and Plant Conservation, Khuan Khanun 93110, Phatthalung, Thailand; by_jai_jung@hotmail.com

**Keywords:** kratom, soil amendments, mitragynine, foliar metabolome, calcium–magnesium ratio, nutrient antagonism

## Abstract

This study investigates how soil calcium (Ca) and magnesium (Mg) supplementation influence mitragynine accumulation in *Mitragyna speciosa* (kratom), addressing the lack of quantitative thresholds in previous research. Seedlings from a uniform seed stock were cultivated in a controlled environment using a standardized soil mix (soil:peat moss:earthworm castings, 6:1:1). Following an initial growth phase, Ca and Mg were applied at three concentrations and in fixed Ca:Mg ratios (5:1, 10:1, 20:1) using gypsum and Epsom salt. Over a 45-day treatment period, growth parameters and mitragynine levels were assessed one week after the final application. Seedlings under control had the highest total biomass (102.35 g), significantly exceeding both the Mg-only and Ca:Mg treatments (64–84 g), and values above the typical upper threshold of 20 did not suppress growth, as evidenced by unchanged root-to-shoot ratios across treatments. In contrast, mitragynine accumulation was the highest under moderate Ca:Mg ratios (8–10), exhibiting a 2–14% increase relative to the control, suggesting that production of this alkaloid is more sensitive to nutrient balance than overall growth. These findings underscore the importance of nutrient ratios, rather than individual nutrient concentrations, in regulating both vegetative development and alkaloid production in kratom. Maintaining an appropriate Ca:Mg ratio can support efficient seedling growth as well as maximizing mitragynine levels. Preliminary field trials over a span of one month indicate that field-grown seedlings exhibit a similar result with high growth and mitragynine content in soils having a Ca:Mg ratio of 1:10. Future studies should test these responses under field conditions and over longer growth periods.

## 1. Introduction

Since the decriminalization of *Mitragyna speciosa* (kratom) in Thailand in 2021, cultivation has expanded rapidly, with over 2100 certified plots recorded nationally by 2022, and a reported average revenue of approximately 145,000 THB per household at a production cost of 3578 THB per rai [1]. The production, ranging from smallholder farms to large-scale operations, caters to both domestic and international markets [2]. The United States currently represents the largest export destination for kratom products, despite regulatory restrictions in several states [3]. Consumption varies from traditional brewed teas and energy-boosting beverages to high-purity extracts, with particular pharmaceutical interest in 7-hydroxymitragynine, a minor but potent alkaloid reputed to possess analgesic properties significantly stronger than morphine, with fewer side effects [4,5]. This diversification has led to a sectorial emphasis on high-yield leaf production for commodity-grade powders, as well as targeted enhancement in alkaloid content for medicinal use.

Commercial cultivation practices are closely aligned with market demand. For high-volume output, growers prioritize bushy plants with abundant low-lying branches, which enable efficient harvesting of the 2nd to 3rd leaf pairs, which are typically the most commercially valuable [6]. Environmental conditions such as soil composition, water availability, and light exposure directly affect leaf yield and phytochemical profile [7]. For example, southern Thailand’s kratom-growing regions experience high solar irradiance (~18 MJ m^–2^day^–1^), cloud cover, and frequent rainfall, which collectively support dense, water-retentive soils [8].

Till date, over 200 alkaloids have been identified in kratom [9], with mitragynine being the most abundant, typically constituting over 66% of total alkaloid content [8]. Other significant compounds include 7-hydroxymitragynine, speciogynine, speciociliatine, mitraciliatine, and corynantheidine, among others [10]. Notably, some alkaloids such as speciociliatine and corynantheidine are more prevalent in younger leaves, hinting at their potential roles in plant defense or response to environmental stressors [11]. Given the variability in alkaloid composition, identifying agronomic practices that stabilize or enhance specific alkaloid concentrations remains a key research priority.

Among the multiple environmental factors affecting alkaloid production, soil nutrient composition is critical. Calcium (Ca) and magnesium (Mg), in particular, have shown consistent positive correlations with mitragynine levels [8,12]. Ca is essential for apical growth, flowering, and seed development, and it enhances nitrogen efficiency [13]. Mg plays a central role in chlorophyll synthesis and enzymatic regulation [14]. Mineral amendments such as gypsum (CaSO_4_) and Epsom salt (MgSO_4_) are preferred as they adjust nutrient profiles without altering pH. For Ca, kratom-growing soils in natural conditions have shown values ranging from 14 to 7079 ppm [12] and 90 to 2654 ppm [8]. Mg levels range from 14 to 1236 ppm and from 9 to 362 ppm, respectively. The calcium-to-magnesium ratio (Ca:Mg) can further modulate nutrient availability and soil structure [15]. While typical ratios fall between 1:1 and 20:1, ranges of 3:1 to 7:1 are often optimal for plant development. While some studies emphasize soil pH over Ca:Mg ratios as primary growth determinants, others highlight the role of Ca-rich soils in improving nitrogen uptake and overall plant vigor [16,17].

Although Ca and Mg are recognized as key factors influencing alkaloid biosynthesis in kratom, quantitative thresholds and optimal Ca:Mg ratios for targeted alkaloid enhancement and agronomic performance remain undefined. Existing studies are largely observational, limiting information that can be used to develop nutrient management protocols specific to alkaloid yield optimization. This study employs a controlled experimental framework to determine the effects of varying the concentration ratios of calcium and magnesium on plant growth and mitragynine accumulation. The broad objectives are to define the ratio thresholds that maximize both biomass and alkaloid output, evaluate the risk of nutrient antagonism or toxicity at elevated calcium levels (4000 ppm), and to identify evidence-based guidelines for Ca and Mg application in commercial kratom production.

## 2. Results

### 2.1. Baseline Soil Chemical Properties Prior to Nutrient Application

As listed in Table 1, the baseline soil mixture had a bulk density of 1.21 g cm^−3^, indicating a moderately compact substrate suited to mechanically support the roots while preserving functional porosity for gas exchange and water retention, both of which are critical for optimal root function and nutrient availability. A volumetric water content of 56% indicates a nearly saturated medium, and is also similar to the natural habitat of kratom, which grows naturally in swamp areas. The soil pH value was 6.54 ± 0.05, while the levels of macronutrients (N, P, K, Ca, Mg, and S) and organic matter content were found to be relatively high, with a Ca:Mg ratio of 13 being significantly elevated compared to the ideal range.

### 2.2. Microclimate in the Greenhouse

**Figure 1:**
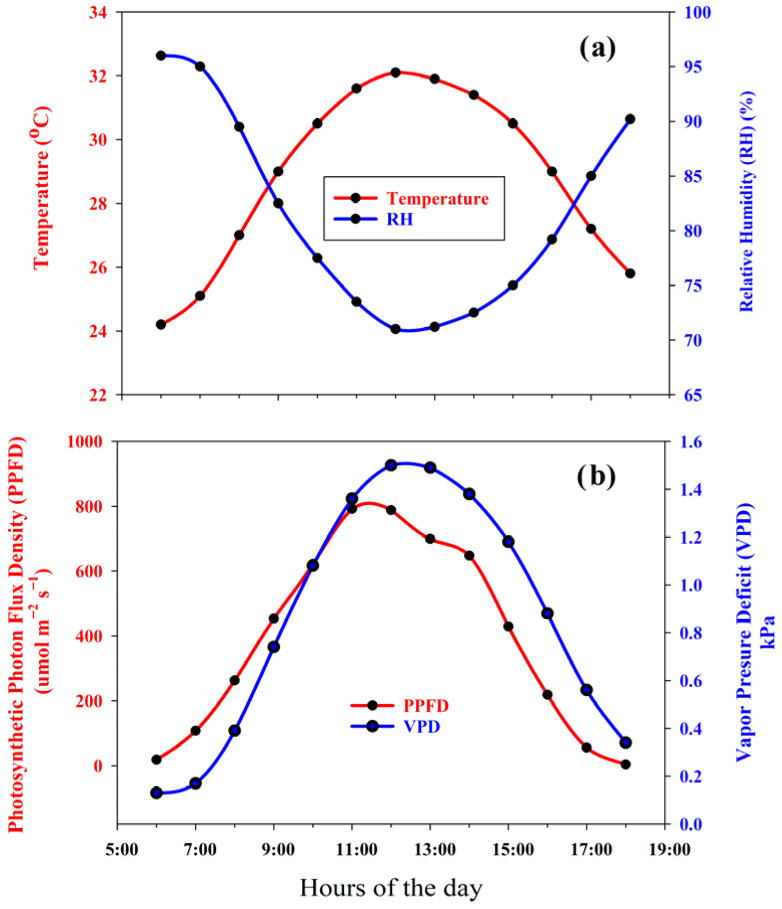
The overall microclimate conditions at the study site in the Phatthalung province of Southern Thailand. (**a**) Air temperature (°C) and relative humidity (%RH) and (**b**) light intensity (PFFD; μmol m^−2^s^−1^) and vapor pressure deficit (kPa).

**Figure 2:**
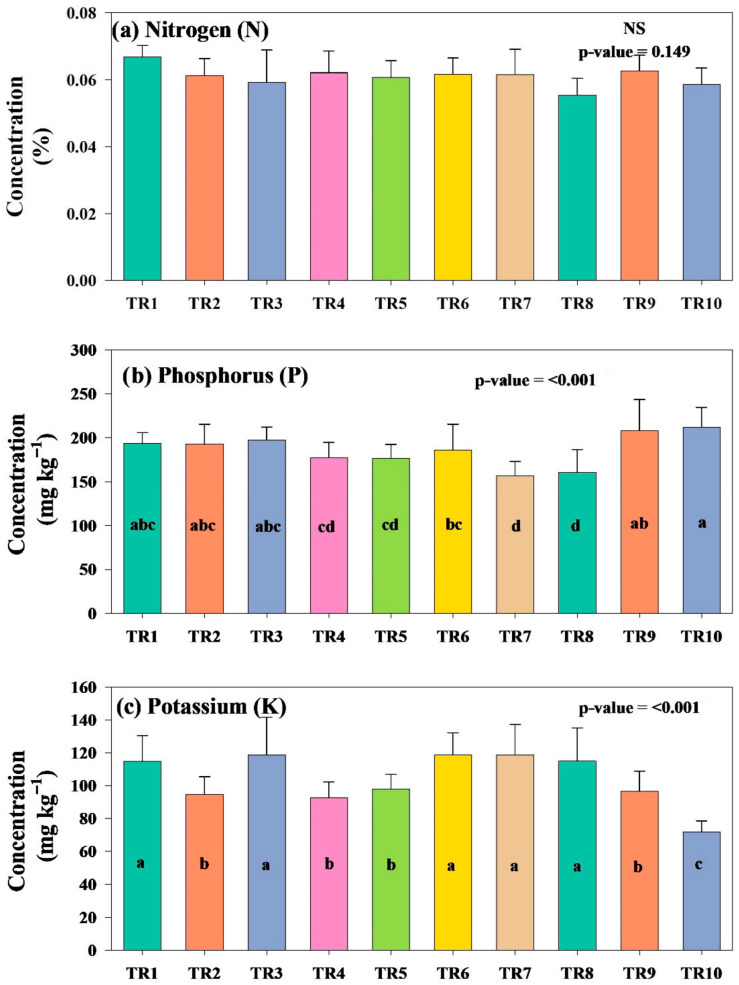
Soil concentrations of nitrogen (N), phosphorus (P), and potassium (K) across all treatment groups (refer to Table 1 and Table 2). Lowercase letters indicate statistically significant differences among treatment means at the 95% confidence level, while “NS” denotes no statistically significant difference.

The hourly diurnal variations in PPFD, air temperature, RH, and VPD are presented in Figure 1. Air temperature gradually increased from the early morning, reaching a maximum of approximately 32 °C around midday, while RH reduced from about 95% in the morning to a minimum of approximately 70% at midday before increasing again in the late afternoon (Figure 2a). PPFD had a typical bell-shaped pattern, rising rapidly after sunrise to peak values of around 800–900 µmol m^−2^s^−1^ near midday and declining toward the evening (Figure 2b). Correspondingly, the VPD increased from low morning values (~0.2 kPa) to a maximum of approximately 1.4–1.5 kPa during midday, reflecting the combined effects of increasing temperature and decreasing RH. The microclimate diurnal conditions were thus characterized by high midday light availability and moderate to high evaporative demand, followed by more humid and cooler conditions during the evening hours.

### 2.3. Soil Chemical Properties After Nutrient Application

**Figure 3:**
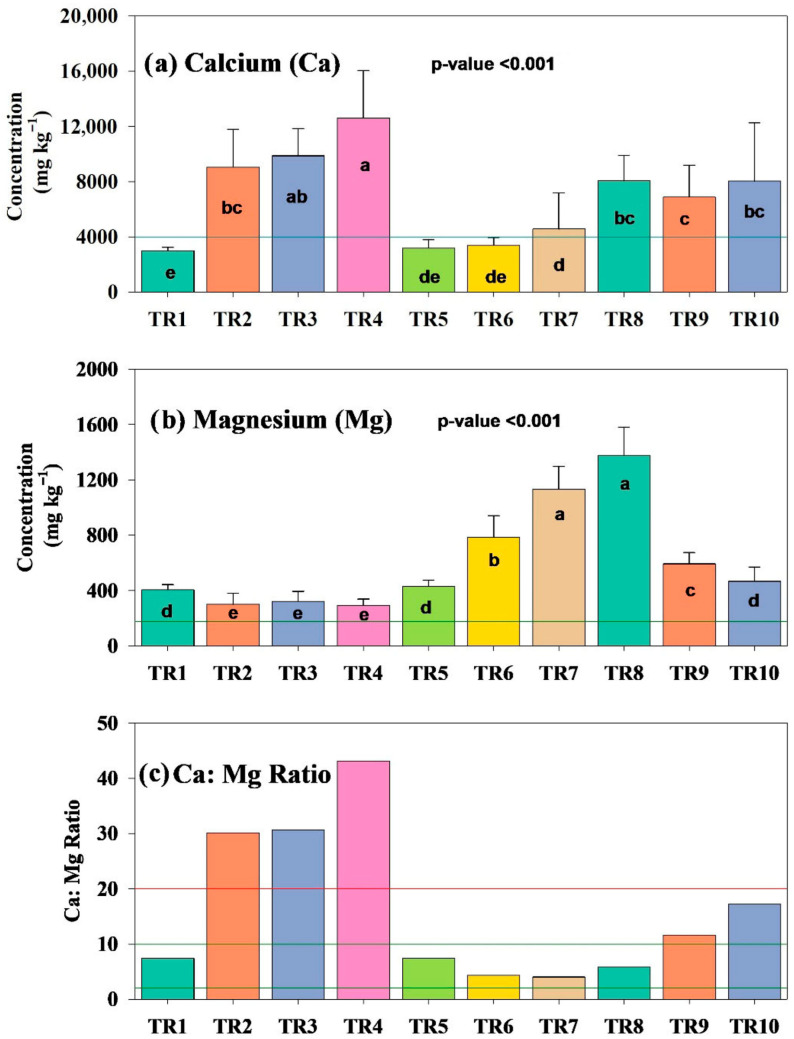
Soil concentrations of calcium (Ca), magnesium (Mg), and the resulting Ca:Mg ratios across treatment groups (refer to Table 2 and Table 3). Lowercase letters indicate statistically significant differences among means at the 95% confidence level. Horizontal green lines represent nutrient levels considered high, while red lines indicate excessive concentrations. In panel (**c**), Ca:Mg ratios exceeding 20 are highlighted as above-normal, with the typical range considered to be between 1 and 20.

Soil chemical properties measured after third-time nutrient application (45 days) are presented in Table 2 and Figure 2 and Figure 3. A comparison between the soil before (Table 1) and after nutrient application (45 days indicated in Table 2) indicates that soil pH increased from 6.54 ± 0.06 to 7.05 ± 0.09, indicating a shift toward more alkaline values resulting from plant growth. Soil pH measured across treatments was similar and remained within a narrow range (6.52 for TR7-7.05 for TR1) across all treatments, with the addition of Ca and Mg lowering the pH relative to values listed in Table 1, but the variations were not statistically different (*p*-value > 0.05). Hence, the observed variations in soil pH did not lead to any disruption in the uptake of gypsum or Epsom salts. This is also evident in Figure 2a, where soil nitrogen levels did not differ significantly across treatments.

Total nitrogen (N) content reduced from 0.26 to 0.07% (73% reduction in values before and after nutrient addition) due to a substantial N uptake by plants. However, the variations (between 0.06 and 0.07%) were not significantly different across treatments post addition, indicating no major effect from external nutrient additions, as seen in Figure 2a. The available phosphorus (P) decreased slightly from 213.40 (pre) to 193.27 ppm (post-addition) (9% reduction), while potassium (K) had a pronounced reduction from 376.80 to 114.68 ppm (70% reduction) before and after the nutrient addition. Post-addition soil samples indicate moderate variability in the levels of P and K, with significantly different variations across treatments (Figure 2b,c). The highest P values were measured for TR9 and TR10, while TR7 and TR8 had the lowest concentration. K concentration was higher in TR1, TR3, TR6, TR7, and TR8, while the lowest concentration was measured in TR10.

Exchangeable Ca decreased by approximately 50%, from 6073.60 to 3011.59 ppm, whereas Mg levels declined slightly from 456.20 to 404.73 ppm (11% reduction). As a result of planting, the Ca:Mg ratio decreased from 13.30 to 7.44 in control conditions (TR1), indicating a more balanced cation relationship. Nutrient addition led to a higher-than-expected and significantly different accumulation of Ca in Ca- and Mg in Mg-only treatments (Figure 3). Calcium additions led to progressively higher Ca concentrations, reaching over 12,000 ppm at the highest application rate (4000 ppm or TR4), and hence led to corresponding reductions in Mg and elevated Ca:Mg ratios (Figure 3a).

**Figure 4:**
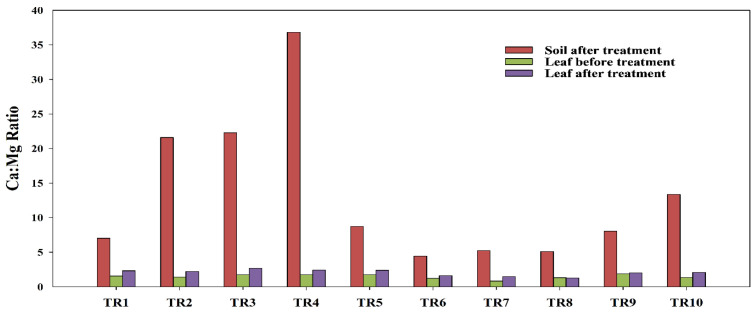
Calcium: magnesium ratio (Ca:Mg ratio) of soil after applying the treatments (red bar), leaf before applying treatments (green bar) and leaf after applying treatments (purple bar) in kratom seedlings.

In contrast, Mg-supplemented treatments exhibited substantial increases in Mg, particularly at 250 ppm (TR7), with concentrations exceeding 1100 ppm, well above the 200 ppm Mg reference threshold (horizontal line in Figure 4), resulting in the lowest measured Ca:Mg ratio (see Figure 3c and Table 2). Treatments with fixed Ca:Mg ratios produced intermediate values, with Ca:Mg 5:1 or TR8 resulting in the highest Mg accumulation (~1378 ppm). The measured Ca:Mg ratios for TR-TR10 (see Table 3) differed from the targeted ratios, likely due to existing Ca and Mg in the base soil mixture, as observed in Table 2. Progressively higher Ca concentrations in the Ca-only supplementation resulted in the measured Ca:Mg ratios exceeding the upper threshold of 20 (marked by a red line in Figure 3c). These shifts in nutrient balance could imply antagonistic interactions between the two cations and confirm that both nutrient concentrations and their ratios are influenced by application rates and base soil composition apart from plant uptake.

### 2.4. Growth Measurements and Leaf Nutrient Concentration

Application of Ca and Mg significantly influenced the RGR of both the stem DBH and H (Table 3). The highest overall growth, including H, DBH, and total dry biomass, was measured in treatments with higher additions of Ca (TR3 and TR4) or for Ca:Mg ratios exceeding 20. Even though the above- and below-ground biomass (fourth and fifth columns of Table 4) was not significantly different, the total biomass differed significantly among treatments, with the control (TR1) and the addition of 500 ppm Mg (TR7) resulting in the highest and lowest values, respectively. No significant variations were observed for the root:shoot ratio between treatments (0.37–0.47), which could be suggestive of balanced biomass allocation in a non-stressful growing environment.

Considering both growth rate and biomass production together, TR3 (Ca 2000 ppm) was identified as the best treatment for overall growth, as it produced the fastest vegetative growth while maintaining acceptable biomass allocation (Table 4). In TR3, the soil pH was measured to be slightly acidic (6.62 ± 0.02), a range that is favorable for the availability of most macronutrients, including P and K. A substantially higher soil Ca concentration likely enhanced cell wall formation, membrane stability, and meristematic activity, thereby supporting a significantly higher relative growth rate of stem DBH and H. Although the Ca:Mg ratio measured for TR3 was markedly elevated (30.69), potentially limiting Mg availability, soil Mg levels remained sufficient (321.52 ± 73.62 ppm) to avoid visible growth suppression. The result suggests that high Ca supply under moderately acidic pH promoted a faster growth in kratom seedlings, even under an imbalance in the Ca:Mg ratio.

Leaf nutrient composition differed significantly both among treatments and between times before and after the application of Ca and Mg (Table 4). Across all treatments, leaf N, P, and K concentrations generally declined significantly after supplementation in most treatments, as indicated by a paired *t*-test (*p*-value < 0.05–0.001), suggesting dilution effects associated with enhanced growth and nutrient rebalancing. In contrast, leaf Ca and Mg exhibited more variable and treatment-specific responses. Leaf Mg increased significantly after amendment in several treatments (notably TR3, TR6, TR7, and TR10), whereas Ca showed pronounced increases in treatments receiving higher Ca availability, resulting in significant treatment effects across columns (*p*-value < 0.001). Notably, treatments associated with elevated Ca accumulation did not consistently correspond to higher Mg uptake, highlighting competitive leaf-level interactions between Ca and Mg. These results indicate that the addition of Ca and Mg altered the nutrient partitioning rather than uniformly increasing nutrient concentrations, with differential regulation of Ca and Mg uptake emerging as a key driver of the subsequent growth and mitragynine accumulation.

Leaf macronutrient composition was significantly different among treatments, with consistently high K levels across all treatments and higher proportions relative to N, Ca, Mg, and P. Leaf N and P remained comparatively similar across treatments, suggesting that growth responses were not limited by N or P availability. Foliar Ca concentrations increased under Ca-only treatments (TR2–TR4), most probably due to the elevated soil Ca availability. In contrast, the variations in Mg levels were relatively small among treatments, indicating partial physiological regulation of Mg uptake despite large differences in soil Ca supply.

A marked increase in soil Ca:Mg ratios was observed under Ca-only treatments, particularly in TR2-TR4 (Figure 4), as followed by nutrient application. However, leaf Ca:Mg ratios ranged between 1.2 and 2.7, remaining substantially lower and less variable relative to variations seen earlier in soil ratios, demonstrating strong homeostatic control of cation balance at the plant level. Despite exceptionally high soil Ca:Mg ratios, TR4 and TR3 had modest increases in leaf Ca:Mg, indicating strong physiological regulation of Ca and Mg uptake. In contrast, even though TR9 and TR10 had moderate soil Ca:Mg ratios, comparatively higher foliar Ca:Mg ratios suggest a more efficient translocation or altered ion balance. This divergence highlights treatment-specific Ca–Mg interactions and underscores that extreme soil Ca enrichment does not necessarily enhance leaf Ca:Mg ratios.

### 2.5. Foliar Mitragynine Quantification

**Figure 5:**
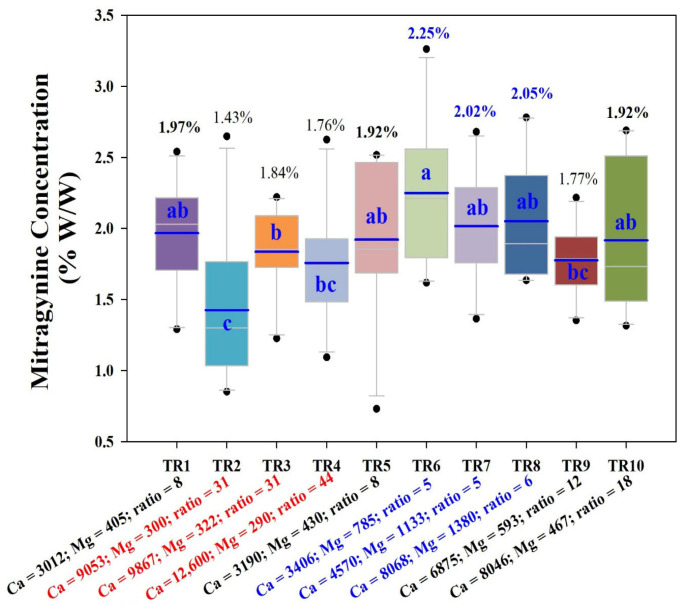
Mitragynine content (% by weight) in kratom seedlings under different nutrient levels, shown as a box plot, with blue horizontal bars indicating mean and white line for median. Different letters indicate statistically significant differences between groups at 95% confidence. The labels on the *x*-axis indicate the concentration levels of calcium and magnesium nutrients, as well as the calcium-to-magnesium ratio measured in the soil. Blue-colored *x*-labels represent the treatment groups with the highest mitragynine content (over 2%), whereas red-colored represent groups with the lowest mitragynine content (ranging between 1.4 and 1.8%).

The effect of different levels of Ca:Mg ratios on the accumulation of mitragynine in kratom seedlings is presented in Figure 5. Statistically significant differences were observed among treatments, with mean mitragynine levels ranging from 1.43% to 2.25% (*p*-value < 0.05). The highest concentrations (above 2%) were measured in TR6, TR7, and TR8, all characterized by moderate Ca:Mg ratios below 6, as highlighted by blue labels on the *x*-axis. In contrast, TR2, TR3, and TR4 had the lowest mitragynine levels (1.43–1.84%), all associated with high measured Ca (9000–12,600 mg·kg^−1^) and low Mg (<350 mg·kg^−1^) concentrations, or elevated Ca:Mg ratios (≥30). Intermediate mitragynine concentrations (~1.92%) were measured in the control (TR1), TR5, and TR10 or in the moderate Mg level and Ca:Mg ratio.

### 2.6. Non-Metric Multidimensional Scaling (NMDS) Constrained Ordination

**Figure 6:**
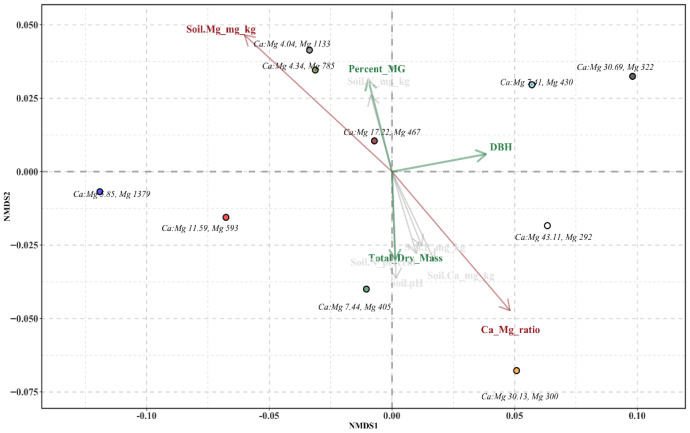
NMDS constrained ordination showing key factors influencing growth and mitragynine content. The stress value was 0.091 with an R^2^ of 0.984, which was below the acceptable threshold of 0.10, indicating good representation of multivariate relationships in two-dimensional space [18]. Growth traits (DBH, Total Dry Mass, Percent MG) are represented by green arrows, soil factors (Soil Mg_mg_kg, Ca:Mg ratio) by red arrows. Arrow direction and length indicate strength and relationship of each factor. Each point represents the treatment groups 1–10 labeled by the Mg levels and Ca:Mg ratios after addition of nutrients to the base soil mixture.

The NMDS constrained ordination highlights distinct associations between soil nutrient parameters and plant performance traits, including mitragynine content, stem DBH, and total dry biomass across treatments, as presented in Figure 6. Among the soil variables, Mg concentration (Soil Mg_mg_kg) and the calcium-to-magnesium ratio (Ca:Mg ratio) were found significant across treatments, as indicated in Table 5. A higher mitragynine content (Percent MG) was aligned with increasing soil Mg and decreasing Ca:Mg ratios, total dry mass was associated with an increasing Ca:Mg ratio, while DBH was not associated with either of the significant soil parameters.

**Figure 7:**
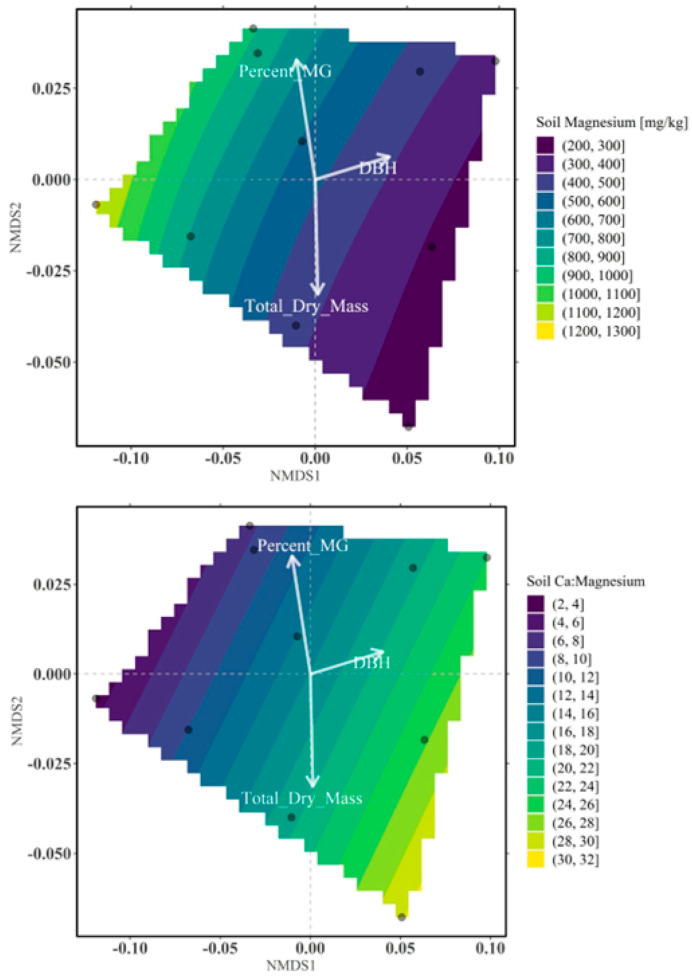
Constrained NMDS ordination contour plots illustrating the relative importance of factors influencing plant growth and mitragynine accumulation, focusing on magnesium (**upper panel**) and the calcium-to-magnesium ratio (**lower panel**). White arrows and vectors represent growth parameters and mitragynine content, while contour lines indicate gradients of magnesium concentration, ranging from low (dark purple) to high (yellow). The optimal levels for promoting growth and mitragynine accumulation correspond to the direction indicated by the vectors.

Figure 7 presents the variations in Mg and Ca:Mg ratio levels depicted as contour gradients, with significant plant growth traits (DBH and total dry mass) and mitragynine accumulation illustrated through vectors. Mitragynine content was highest at elevated Mg concentrations, ranging from 800 to 900 mg kg^−1^ (ppm), combined with a Ca:Mg ratio between 8 and 10. In contrast, biomass accumulation and overall plant growth were observed at lower Mg levels, approximately 400–500 mg kg^−1^ (ppm). However, when Ca availability was sufficiently high, as indicated by an elevated Ca:Mg ratio to values between 20 and 22, favorable growth responses were also detected. This condition corresponds to soil Ca concentrations in an approximate range of 8000–12,000 mg kg^−1^ (ppm).

### 2.7. Preliminary Field Testing

#### 2.7.1. Soil Parameters After Ca:Mg 1:10 Ratio Application

After a month of treatment, Ca:Mg supplementation resulted in shifts in exchangeable cation composition relative to the control (Table 6). However, as previously observed, soil pH remained moderately acidic in both groups, ranging from approximately 5.0 to 5.9 in the treated soils and 5.5 to 6.6 in the control, indicating that gypsum and Epsom salt application did not substantially alter the soil pH after a month. Likewise, soil organic matter, total carbon, and total nitrogen were comparable between treatments, suggesting that the amendment primarily influenced exchangeable base cation composition rather than broader soil fertility. In contrast, pronounced differences were observed in the exchangeable Ca concentration and subsequently the Ca:Mg ratio. Treated soils contained substantially higher exchangeable Ca (approximately 2200–7820 mg kg^−1^) relative to control (680–927 mg kg^−1^). Exchangeable Mg concentrations were generally similar between groups, although slightly lower values were noted in some treated soils, likely reflecting the relative increase in Ca abundance. As a result, the Ca:Mg ratio increased markedly in the amended soils, ranging from 2.3 to 8.6, whereas the ratio in control soils remained below 1.0 (0.71–0.87), consistent with a Mg-dominant exchange complex in the untreated condition.

#### 2.7.2. Relative Growth Rate and Mitragynine Content

Supplementation at a Ca:Mg ratio of 10:1 significantly promoted kratom seedling growth relative to the control (Table 7). After a month, the relative height growth rate reached 0.052 ± 0.016 m and the relative diameter growth rate reached 0.151 ± 0.080 cm, both significantly higher than in the control treatment (0.016 ± 0.013 m and 0.018 ± 0.013 cm, respectively; *p*-value < 0.05). Seedlings receiving 10:1 Ca:Mg treatment exhibited a significantly higher foliar mitragynine concentration (0.82 ± 0.13% dry weight) than control seedlings (0.68 ± 0.12% dry weight; *p*-value = 0.040).

## 3. Discussion

The results of this study demonstrate that mitragynine accumulation is relatively more sensitive to the balance between Ca and Mg than to either nutrient added in isolation, a finding that extends prior observational work [8,12] by providing quantitative thresholds under controlled conditions. In addition, soil nutrient levels did not increase in direct proportion to nutrient inputs, likely because the growth medium already contained background Ca and Mg, and the actual Ca:Mg ratios therefore differed from the intended treatments, with Ca-only treatments exceeding the agronomic threshold of 20 [19]. Three consistent patterns emerged, as nutrient accumulation in soil did not scale with input due to non-zero baseline levels of these nutrients, and seedlings maintained stable growth even at Ca:Mg ratios exceeding commonly cited thresholds [16].

### 3.1. Soil Evaluation Pre- and Post-Supplementation

Thailand’s dominant soil type poses a challenge, as it is characteristically acidic (pH < 5) [20], nutrient-deficient (especially in Ca and K), and rich in iron/aluminum oxides [21]. Ideal nutrient uptake generally occurs at a pH between 6.2 and 7.3 [22], a range uncommon in native soils. Moreover, nutrient interactions are complex; for example, nitrogen and phosphorus uptake are interdependent [23], and microbial associations [24] such as mycorrhizae influence nutrient and water availability. The Ca:Mg ratio of 13:1 was significantly higher than the ideal ranges are often reported to be—5:1 to 8:1 [16].

Five weeks after the final nutrient application, soil pH was measured between 6.5 and 7.0 across treatments, with slight yet insignificant reduction in pH (compared to pre-supplementation). This suggests that the added salts may have influenced soil acidity through changes in cation-exchange dynamics or nutrient solubility, probably due to marginal acidifying tendencies of gypsum [15,25] and Epsom salts [26]. The soil pH range measured post-treatment (6.52–7.05) was within the optimal zone for Ca and Mg solubility (roughly pH 6.0–7.5), which implies that even with slight acidification, neither nutrient level approached the solubility threshold that restricts uptake. Previous studies [27,28] report pH-dependent restriction of nutrient solubility primarily at more extreme pH values, particularly below 5.5 or above 8.0, where mineral precipitation or charge-mediated exclusion becomes significant. Gypsum and Epsom salts used in the current study are highly soluble sulfate salts that dissociate readily across the measured pH range, unlike carbonates or phosphate-bound forms that are pH-sensitive. Furthermore, a moderately high organic matter content (95.62 g kg^−1^) and cation exchange capacity likely buffered pH changes while simultaneously providing additional exchange sites to retain Ca^2+^ and Mg^2+^. As such, a combination of soluble salt forms, moderate pH, and high organic matter might explain why accumulation was not restricted, in contrast to studies that used carbonate or oxide-bound nutrient sources in more acidic or alkaline soil systems. Post-treatment soil analyses indicate pronounced nutrient accumulation in Ca-only and Mg-only treatments, with Ca and Mg levels far exceeding the respective application thresholds of 4000 ppm for Ca and 200 ppm for Mg (Figure 2, Table 2). This higher-than-expected buildup could reflect over-retention in the soil matrix or a plant-level self-regulatory response to reduce uptake efficiency under abundance of Ca availability [29,30].

Ca antagonism is known to restrict the uptake of several essential nutrients, including nitrogen, phosphorus, potassium, magnesium, as well as iron, boron, zinc, copper, and manganese through cation competition and reduced availability at the root surface [29,31,32,33]. A drop in soil nitrogen across all treatments relative to the baseline values was likely due to plant uptake rather than treatment-specific effects, while insignificant variations in nitrogen levels across treatments is supported by a previous observation that soil nitrogen is primarily driven by variations in soil pH [34]. In contrast, the levels of phosphorus and potassium were significantly different, reinforcing the sensitivity of these nutrients to calcium and magnesium supplementation [32] or a consequence of various factors and equilibrium reactions [35,36,37].

Kratom seedlings grown under elevated Ca conditions maintained stable growth across all measured parameters. Variation in root-to-shoot ratio was not significantly different across all treatments, indicating similar biomass allocation and no evidence of shifts in carbon partitioning related to physiological stress [38]. This was even the case for soil Ca:Mg ratios above 20, which are typically considered inhibitory in many cropping systems [19]. Under non-limiting or mildly variable nutrient conditions, plants tend to maintain allometric stability in biomass partitioning, prioritizing structural growth over adaptive reallocation [38]. A shift towards greater root allocation tends to occur under nutrient deficiency (as roots proliferate to forage for scarce resources) or toward shoot allocation under nutrient abundance (to maximize light capture). The absence of significant variation observed in the current study suggests that neither nutrient deficiency nor toxicity reached a threshold sufficient to trigger adaptive reallocation, consistent with the observation that leaf Ca:Mg ratios remained tightly regulated (1.2–2.7) regardless of soil extremes, indicating effective homeostatic control. Furthermore, Ca plays a structural role in root elongation and cell wall rigidity [39,40], with sufficient Ca availability across all treatments (even the control had 3011 ppm post-treatment) sustaining root growth uniformly. Mg, while central to metabolic function, did not appear to reduce to levels severe enough to impair root development in any treatment. The stable root:shoot ratio therefore reflects the combination of adequate baseline nutrient levels in the soil mix and the plant’s strong internal cation regulation.

However, leaf Ca:Mg ratios remained tightly regulated, ranging from 1.2 to 2.7, despite soil ratios exceeding well beyond threshold values, indicating that kratom seedlings were able to actively maintain internal cation balance. Furthermore, the foliar Ca:Mg ratios observed in this study (1.2–2.7) are comparable to those reported for other species. Dominant tree species in Hainan’s coastal forests exhibit ratios within the optimal 1–7 range [41], in apple leaves between 2.0 and 2.6 [42], while in *Brassica rapa*, they range from 0 to 2.0 depending on exogenous supply [43].

Elevated Ca concentrations can promote the precipitation of Ca-phosphate minerals (e.g., hydroxyapatite, dicalcium phosphate), reducing plant-available P, which is a documented antagonistic interaction [32]. Conversely, Mg supplementation may have indirectly improved P availability by maintaining soil pH in the near-neutral range optimal for P solubility. Ca^2+^ and Mg^2+^ compete with K^+^ for cation exchange sites on soil colloids, and elevated divalent cation concentrations can displace K from exchange sites, reducing its availability or increasing leaching [35,44]. The variation across treatments therefore reflects a combination of competitive displacement of K^+^ by Ca^2+^ and Mg^2+^ at cation-exchange sites, pH-mediated changes in P precipitation and solubility, and differential plant uptake driven by treatment-specific growth rates.

These values place kratom within the range observed for other woody species, suggesting similar Ca–Mg balance requirements. In conclusion, Ca-only treatments lead to a concentration-dependent buildup of soil Ca and a corresponding decline in Mg, which elevates the respective Ca:Mg ratios above 40, with subsequent implications for nutrient availability and antagonism [33]. In contrast, Mg-only treatments cause substantial increases in soil Mg, resulting in the lowest Ca:Mg ratio. Thus, in soils with higher levels of Ca, addition of Mg can rebalance nutrient ratios without substantially acidifying the soil. This observation is further accentuated through similar observations from a month of field trials with Ca:Mg supplementation rapidly altering soil cation balance, primarily by increasing exchangeable Ca and shifting the Ca:Mg ratio upward, while leaving pH and bulk soil organic properties largely unchanged.

### 3.2. Effect on Plant Physiology

Ca and Mg have contrasting regulatory roles at the physiological level, with Ca being critical for membrane stabilization and structural integrity [30,45,46], whereas Mg is central to metabolic function, acting as a counter-ion in thylakoid membranes and as an essential cofactor for ATP-dependent reactions [30,45]. As a result, Ca dominance can favor structural growth while simultaneously imposing a functional Mg limitation that constrains metabolic processes. Plant growth alone alters the soil cation balance, with exchangeable Ca reducing by about 50% and Mg decreasing slightly under control conditions, suggesting that kratom seedlings drew down Ca more rapidly, possibly due to its role in cell wall structure or root development [47].

A larger variance was observed in leaf Ca and Mg levels, given their central roles in ionic balance and structural function [30]. A proportionate Mg accumulation in the leaves did not scale with soil Mg availability under high-Ca treatments, underscoring antagonistic interactions at the root–soil interface, where excess Ca could constrain Mg uptake despite adequate soil supply [48]. The Ca–Mg antagonism, observed in the current study, can manifest through opposing structural and metabolic controls, and is well-documented in plant nutrition, in which elevated levels of one cation suppresses the uptake or physiological effectiveness of the other, even when soil concentrations are not limiting [30,49]. These include a wide range of plant species and soil systems, where high Ca availability can reduce Mg uptake and utilization through competition for exchange sites and transport pathways [44,50,51,52]. The contrasting measurements of foliar macronutrients point to a clear decoupling between growth-driven soil nutrient dilution and cation regulation. Foliar nitrogen and phosphorus concentrations reduced slightly after supplementation, likely due to dilution effects from increased biomass [53].

This divergence between soil and leaf Ca:Mg ratios further suggest that kratom prioritizes internal cation balance over passive uptake, a trait that may ultimately shape the plant’s metabolic output under variable soil conditions and contribute to consistent secondary metabolite regulation. The absence of altered biomass partitioning or growth suppression under these conditions indicates that kratom seedlings can tolerate higher levels of Ca in the soil without detectable impairment, consistent with calcium’s structural roles in cell wall integrity and root elongation [39,40]. These results indicate that nutrient additions, rather than plant demand, determine post-treatment soil chemistry, and that both nutrient levels and their ratios are strongly modulated by initial substrate composition.

### 3.3. Mitragynine Accumulation

The biosynthesis of mitragynine occurs through the monoterpene indole alkaloid pathway. Recent biochemical and enzymatic studies of mitragynine and related alkaloids demonstrate that pathway flux and stereochemical outcomes are sensitive to upstream metabolic conditions, supporting the view that nutrient-driven shifts in metabolic balance can substantially influence alkaloid profiles without direct inhibition of biosynthetic enzymes [54,55].

The contrasting roles observed in soil and foliar accumulation of Ca and Mg extend to secondary metabolism and alkaloid regulation. It has been previously reported that at the signaling level, Ca participates in the regulation of monoterpene indole alkaloid biosynthesis in *Catharanthus roseus* [56,57]. The authors observed that distinct Ca release pathways differentially regulate alkaloid accumulation, highlighting that Ca effects are context-dependent and tightly linked to cellular compartmentalization and signaling dynamics [56,57]. Ca has been shown to strongly inhibit the biosynthesis of isoquinoline alkaloids in *Papaver somniferum*, primarily by stabilizing membranes, suppressing proton ATPase activity, and reducing substrate availability for alkaloid-synthesizing enzymes, thereby limiting active transport within highly compartmentalized biosynthetic pathways [58]. In contrast, through its involvement in photosynthesis, Mg supports metabolic capacity, carbon assimilation, and energy transfer, with Mg fertilization associated with enhanced metabolic activity and altered secondary metabolite profiles in medicinal plants [59,60].

Mitragynine accumulation was higher in treatments with higher Mg availability and Ca:Mg ratio in the soil rather than with absolute Ca concentration, suggesting that secondary metabolism was influenced by the combined effect of Ca and Mg rather than by absolute nutrient abundance reported in chamomile (*Matricaria recutita* L.) [61], independent of the vegetative growth. One possible explanation could be in the biochemical role of Mg as a cofactor in numerous enzymatic reactions and a stabilizer of nucleotide structures [62,63], and its involvement in the metabolic pathways leading to production of alkaloids [64] including mitragynine. Mitragynine biosynthesis proceeds from strictosidine through a series of post-strictosidine modifications [55,65], involving sequential reduction and methylation from strictosidine, followed by indole 9-hydroxylation and C-9 O-methoxylation, with early reactions occurring in roots and terminal modifications associated with leaf-specific mitragynine accumulation.

Within this post-strictosidine framework, a recent study reported that foliar Mg status is positively associated with monoterpene indole alkaloid biosynthesis in *C. roseus* via strictosidine through multiple post-strictosidine enzymatic modifications [66]. Mg has a role in sustaining the metabolic capacity required for alkaloid modification and accumulation. Being a central atom of chlorophyll [67], Mg supports photosynthetic efficiency by stabilizing light capture and electron transport, thereby providing the carbon supply, reducing power, and metabolic energy required for reductive, hydroxylation, and methylation reactions characteristic of post-strictosidine alkaloid biosynthesis. Consistent with previous observations, the authors conclude that sufficient Mg availability supports core primary metabolic functions responsible for alkaloid accumulation in medicinal plants, such as photosynthetic carbon assimilation, energy transfer, and amino acid biosynthesis [68].

In contrast, increased Ca supplementation is associated with a reduction in mitragynine levels, indicating that excessive Ca may disrupt key physiological or biochemical pathways required for alkaloid biosynthesis, possibly by antagonizing Mg uptake [69]. Such antagonistic nutrient interactions can be driven by imbalance in supplementation [70], with high Ca availability reducing Mg uptake by plants through competitive interactions at the root interface [30,71]. Such antagonism has been reported to influence carbon partitioning and resource allocation to metabolism rather than simply limiting growth [30].

The combined soil and plant data indicate that high mitragynine content is associated with higher soil Mg levels (800–900 ppm) and moderate Ca:Mg ratios (8–10), which results in leaf Ca:Mg ratios between 1.2 and 2.7, reflecting strong physiological regulation. This would indicate that, while Ca availability primarily drives vegetative growth as reported in coffee [72], Mg availability and ionic balance help regulate alkaloid biosynthesis as observed in mulberry [73]. This stable biomass allocation across treatments suggests that mitragynine biosynthesis may be influenced by carbon–nitrogen resource allocation that balances growth with secondary metabolite accumulation [74] under non-stressful conditions. Although mitragynine content in young kratom plants is generally reported to remain below 1%, the values measured after one month of field trial indicate that balanced Ca and Mg supplementation can enhance alkaloid accumulation and improve the production of the secondary metabolite in kratom seedlings.

### 3.4. Limitations

This study shows that Mg and Ca:Mg ratios influence both kratom growth and mitragynine yield, with nutrient balance and not individual levels being the key driver of plant performance and alkaloid production. However, a few limitations of the current study should be acknowledged. The experiment was conducted under controlled conditions on early vegetative growth, which may not fully reflect responses across the plant’s full life cycle or under field conditions with abiotic and biotic influences [6,7,8,75] as well as associations with environmental variables [76]. The role of other essential micronutrients was not addressed, and only mitragynine was quantified, despite kratom’s chemically diverse alkaloid profile. Future research should expand on these results by evaluating long-term effects of nutrient ratios across developmental stages, including flowering and harvest, and by incorporating soil microbiome dynamics and multi-alkaloid profiling. Such studies will help refine nutrient management strategies that optimize both biomass and phytochemical output in commercial kratom production.

## 4. Materials and Methods

**Figure 8:**
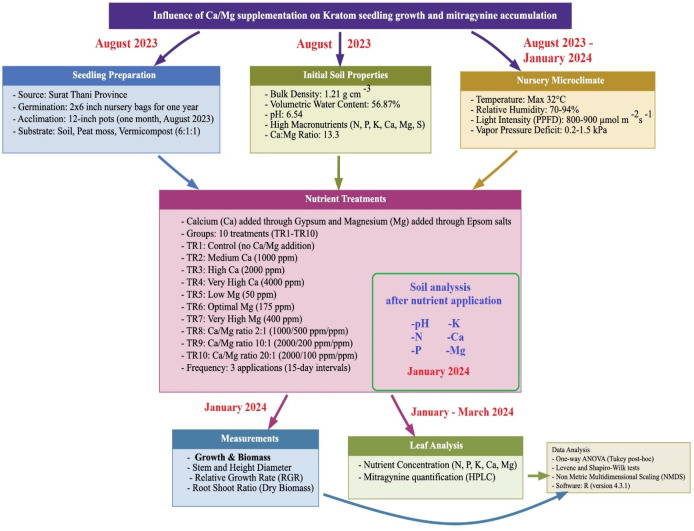
Schematic of the workflow and timeline of the experimental design to assess the effects of calcium (Ca), magnesium (Mg), and Ca:Mg ratios on kratom (Mitragyna speciosa) seedling growth and mitragynine accumulation.

The workflow of the study, conducted between August 2023 and March 2024 at a greenhouse site in Phatthalung province, is presented in Figure 8. The various steps are presented in the following subsections below.

### 4.1. Seedling Preparation and Greenhouse Conditions

Seeds were collected from healthy kratom trees that were free from visible diseases and pests and originated from similar genetic sources (Surat Thani province, 180 km away from the nursery) in order to minimize genetic variation among seedlings. Using seeds from a single or closely related genetic background reduces variability compared with seedlings obtained from multiple unknown sources, while still maintaining the diversity of the seedlings. The seeds were sown in 2 × 6-inch nursery bags filled with local soil and placed in a nursery greenhouse at the study site. The nursery was set up in a shaded greenhouse to reduce excessive light stress. An automated environmental monitoring system was installed that continuously monitored light intensity (measured through photosynthetic photon flux density (PPFD)), air temperature, relative humidity (RH), and vapor pressure deficit (VPD) at 10 min intervals throughout the experiment. Seedlings were irrigated twice daily at 08:00 and 16:00 local time (LT) to ensure adequate water availability.

After one year, the seedlings of uniform size and vigor, with heights ranging from approximately 30–50 cm, were transferred in 12-inch diameter pots to acclimatize to the new growing environment prior to subsequent experimental treatments in August 2023. Each pot was filled with an identical soil mass and bulk density to ensure uniform root growth conditions. The mixture that formed the topsoil was purchased from a single source and thoroughly mixed with peat moss (to improve soil porosity) and vermicompost in a ratio of 6:1:1. To achieve an optimal soil bulk density of approximately 1.4 g cm^−3^, suitable for root penetration, aeration, and water retention [77], each pot was filled with approximately 7 kg of the prepared soil mixture. After transplanting and allowing the seedlings to acclimatize to the new environment for a month, the physical and chemical properties of the soil mixture were measured. Five replicates of randomly selected pots were used to determine the baseline properties and nutrient levels of the soil mixture. Soil pH and nutrients were collected from a disturbed sample and analyzed in the Laboratory of Soil, Faculty of Forestry, Kasetsart University.

### 4.2. Nutrient Application

Ca and Mg were supplied in nutrient forms of gypsum (CaSO_4_·2H_2_O, containing 23% Ca) and Epsom salts (MgSO_4_·7H_2_O, containing 10% Mg), respectively. Dosage calculations were based on the target nutrient concentrations for a 7 kg soil weight per pot. For example, to achieve a Ca concentration of 4000 ppm, 28 g of elemental calcium was required, which corresponds to 121.74 g of gypsum. Similarly, a Mg concentration of 400 ppm required 2.8 g of elemental magnesium, equating to 28 g of Epsom salts. Fifteen seedling plots were assigned to be replicates in each treatment (Table 8) for a total of 150 plants. The nutrient treatments were applied 3 times over the 15-day interval during December 2023 to January 2024. After the final application, seedlings were allowed to acclimatize and absorb the nutrients for seven days. Subsequently, a composite soil sampling was conducted using five replicates (pots) from each treatment for a total of 50 samples to analyze soil chemicals after which the physiological measurements and harvesting for growth measurements were conducted.

### 4.3. Growth Measurements and Nutrient Concentration in Leaves

Baseline plant growth measurements included stem diameter (DBH) (taken 20 cm above the soil surface using a Vernier caliper) and plant height (H) which were conducted at an interval of five months (i.e., in August 2023 and then again in January 2024) for all pots. Relative growth rate (RGR) was calculated to compare seedling growth in each treatment following Equation (1):

(1) $$\mathrm{RGR}\;(\mathrm{mm}/\mathrm{cm}\;\mathrm{per}\;\mathrm{month})\;=\;\frac{{\mathrm{Ln}\;(\mathrm{DBH},\;H)}_{\mathrm{month}5}\;-\;{\mathrm{Ln}\;(\mathrm{DBH},\;H)}_{\mathrm{month}1}}{5}$$

Afterward, dry biomass was estimated using five seedlings per treatment group by harvesting the plants, rinsing them with water, and separating the root and shoot portions. Samples were oven-dried at a temperature of 80 °C for 72 h and weighed using 4-digit digital balance. The root–shoot ratio was calculated by dividing the dry biomasses of roots and shoots. The nutrient levels in the sampled leaves were quantified through collection both before and after the nutrient application. Two leaves (per plant) were collected and pooled into three composite samples per treatment (30 samples in total) prior to nutrient application, while the same nutrients were measured after nutrient application in eight samples (80 samples in total). The samples were analyzed for N, P, K, Ca, and Mg levels in the Laboratory of Soil, Faculty of Forestry, Kasetsart University. Leaf N was determined by the Kjeldahl digestion method; P by the vanadomolybdate colorimetric method; and K, Ca, and Mg by atomic absorption spectrophotometry (AAS-flame), following standard analytical protocols [79].

### 4.4. Foliar Mitragynine Quantification

To quantify the mitragynine content, two leaves (from the 2nd and the 3rd pair) per plant were collected and pooled into ten composite samples per treatment (100 samples in total), followed by analysis via High-Performance Liquid Chromatography (HPLC) as elaborated by Leksungnoen et al. [6]. Briefly, leaves were air-dried, ground, and sieved to less than 0.5 mm. Fifty milligrams of each sample was extracted in 5 mL methanol, sonicated for 10 min, incubated for 24 h, and centrifuged at 25 °C and 4500 rpm for 5 min. The supernatant was diluted 1:10 with methanol, filtered through a 0.22-μm PTFE syringe filter, and analyzed via HPLC using an Agilent 1260 system with an Inertsil ODS-3 column (5 µm, 150 × 4.6 mm). The system operated at 226 nm, 27 °C, and 1 mL/min flow rate. Quantification was based on a standard curve generated using a mitragynine reference standard (97.2% purity).

### 4.5. Preliminary Field Experiment

Twenty kratom seedlings, propagated from seeds of the same origin as those used in the pot experiment, were established in the field after they reached approximate height of 100 cm. Based on the optimal Ca:Mg ratio identified in the pot experiment previously, Ca and Mg supplementation was applied at a ratio of 10:1 using gypsum and Epsom salts. After one month, ten seedlings were assigned to the supplementation treatment while the remaining seedlings served as controls. Application rates were calculated for the soil volume surrounding each plant using a circular band positioned approximately 10 cm from the plant base, covering a radius of 10 cm and a soil depth of 15 cm, corresponding to an estimated soil volume of 4712 cm^3^. Assuming an average soil bulk density of 1.4 g cm^−3^, the treated soil mass was estimated at 6.60 kg per plant. To achieve the target supplementation levels, the required elemental inputs were calculated as 59.4 g Ca plant^−1^ and 5.9 g Mg plant^−1^, equivalent to approximately 255 g gypsum plant^−1^ and 60 g Epsom salt plant^−1^, respectively, based on the elemental composition of the fertilizer sources. The fertilizers were evenly distributed within the circular band and added to the soil to a depth of approximately 15 cm. A month after application, relative growth rate and mitragynine content were determined using the same procedures described in Section 4.3 and Section 4.4.

### 4.6. Statistics and Data Analysis

One-way Analysis of Variance (ANOVA) was conducted to assess the effect of Ca, Mg, and their ratios on plant growth metrics, physiological responses, nutrient uptake, and mitragynine content across the ten treatment groups. Homogeneity of variances was verified using Levene’s test, and data normality was tested via the Shapiro–Wilk test prior to ANOVA. Levene’s test was used to confirm the homogeneity of variances across treatment groups for all primary response variables (*p*-value > 0.05 for RGR DBH, RGR Height, total biomass, and mitragynine content). The Shapiro–Wilk test was used to determine the normality of data (*p*-value > 0.05) and any variables showing minor deviations from normality were verified to have residuals within acceptable limits. Differences were deemed significant at *p*-value ≤ 0.05, and Tukey’s Honestly Significant Difference (HSD) post hoc test was used for pairwise comparisons to identify specific treatment effects [80]. Non-Metric Multidimensional Scaling (NMDS) was used to determine the effect of environmental and soil variables on leaf traits, based on Bray–Curtis distances [81] via the metaMDS function in the vegan package [82]. Ordination fit was confirmed through stress values below 0.10. Variable significance was tested using the envfit function with 10,000 permutations (*p*-value ≤ 0.05) and visualized in a triplot showing vectors for environmental factors, leaf traits, and sample locations. All the analyses were performed in R [83].

## 5. Conclusions

This study establishes a controlled experimental framework to determine how varying levels and ratios of calcium (Ca) and magnesium (Mg) influence seedling growth and mitragynine content in *Mitragyna speciosa* (kratom). Despite Ca:Mg ratios exceeding the typical upper threshold of 20, seedlings exhibited stable growth with insignificant differences in root-to-shoot ratios. However, mitragynine accumulation was highest under moderate Ca:Mg ratios (8–10) and higher Mg availability, indicating that alkaloid accumulation is more sensitive to soil nutrient balance than plant growth. These findings highlight nutrient ratios, not individual concentrations, as important to balance both biomass production and alkaloid biosynthesis in kratom, with TR3 (Ca 2000 ppm) producing the highest relative growth rates for stem diameter and height, while TR6–TR8 (moderate Ca:Mg ratios ≤ 6) resulting in the highest mitragynine content. The results can be of practical importance in tailoring nutrient-specific guidelines to optimize both agronomic yield and phytochemical output. Future research should extend these results to field conditions, incorporate additional macro- and micronutrient interactions, and assess nutrient effects across the full life cycle. Integrating these findings with environmental and economic factors will be critical for establishing sustainable, high-quality kratom cultivation systems.

## Figures and Tables

**Table 1:** Physical and chemical characteristics of soil mixture in the pot prior to treatment application.

Soil Property	Values
Bulk Density (g cm^−3^)	1.21 ± 0.01
Volumetric Water Content (VWC) (%)	56.24 ± 8.07
pH (unitless)	6.54 ± 0.06
Organic Matter (OM) (g kg^−1^)	95.62 ± 5.36
Nitrogen (N) (%)	0.26 ± 0.01
Phosphorus (P) (ppm)	213.40 ± 10.60
Potassium (K) (ppm)	376.80 ± 12.40
Calcium (Ca) (ppm	6073.60 ± 1144.20
Magnesium (Mg) (ppm)	456.20 ± 34.62
Sulfur (S) (ppm)	890.73 ± 42.59
Calcium-to-Magnesium Ratio (Ca/Mg)	13.30 ± 2.31

**Table 2:** Soil pH and Ca:Mg ratio after five weeks after nutrient amendments (to alter the calcium or Ca, magnesium or Mg, and Ca:Mg values). Values represent post-treatment concentrations of key macronutrients and micronutrients across all treatment groups, measured to determine the nutrient accumulation and availability following calcium and magnesium supplementation.

Treatment	pH	Ca:Mg Ratio
TR1 (Control)	7.05 ± 0.09	7.44
TR2 (Ca 1000 ppm)	6.65 ± 0.07	30.13
TR3 (Ca 2000 ppm)	6.62 ± 0.02	30.69
TR4 (Ca 4000 ppm)	6.60 ± 0.04	43.11
TR5 (Mg 50 ppm)	6.72 ± 0.03	7.41
TR6 (Mg 175 ppm)	6.56 ± 0.1	4.34
TR7 (Mg 250 ppm)	6.52 ± 0.2	4.04
TR8 (Ca:Mg 5:1)	6.59 ± 0.18	5.85
TR9 (Ca:Mg 10:1)	6.70 ± 0.13	11.59
TR10 (Ca:Mg 20:1)	6.73 ± 0.09	17.22

**Table 3:** Relative growth in stem diameter, height, and biomass of kratom seedlings grown under greenhouse conditions across different nutrient treatments. Values are presented as mean ± standard deviation. Lowercase letters indicate statistically significant differences in means among treatments at the 95% confidence level (*p*-value < 0.05); “NS” denotes no significant difference (*p*-value > 0.05). RGR is relative growth rate; DBH is stem diameter, ABG is above-ground biomass, BG is below-ground biomass.

Treatment	RGR DBH (cm/week)	RGR Height (m/week)	ABG (g)	BG (g)	Total Biomass (g)	Root:Shoot Ratio
TR1 (Control)	0.026 ± 0.004 bc	0.022 ± 0.008 bcd	69.56 ± 16.5	32.79 ± 12.64	102.35 ± 26.71 a	0.47 ± 0.11
TR2 (Ca 1000 ppm)	0.026 ± 0.009 bc	0.026 ± 0.005 abc	61.31 ± 15.8	22.67 ± 4.33	83.98 ± 19.69 ab	0.38 ± 0.04
TR3 (Ca 2000 ppm)	0.036 ± 0.008 a	0.029 ± 0.006 a	51.29 ± 9.7	19.12 ± 4.23	70.41 ± 13.4 b	0.37 ± 0.04
TR4 (Ca 4000 ppm)	0.033 ± 0.003 ab	0.028 ± 0.005 ab	57.99 ± 12.68	26.35 ± 4.09	84.34 ± 13.9 ab	0.45 ± 0.11
TR5 (Mg 50 ppm)	0.032 ± 0.007 ab	0.032 ± 0.007 a	49.41 ± 4.52	22.02 ± 5.61	71.43 ± 9.01 b	0.45 ± 0.09
TR6 (Mg 175 ppm)	0.023 ± 0.005 cd	0.018 ± 0.008 de	53.08 ± 5.59	22.92 ± 2.98	76 ± 7.84 b	0.43 ± 0.04
TR7 (Mg 250 ppm)	0.020 ± 0.007 cde	0.021 ± 0.005 cde	45.53 ± 7.79	19.26 ± 3.42	64.8 ± 8.50 b	0.42 ± 0.10
TR8 (Ca:Mg 5:1)	0.013 ± 0.005 e	0.020 ± 0.007 cde	51.76 ± 9.19	22.94 ± 3	74.7 ± 11.58 b	0.44 ± 0.05
TR9 (Ca:Mg 10:1)	0.017 ± 0.006 de	0.014 ± 0.005 e	51.58 ± 11.1	24.37 ± 8.59	75.95 ± 17.84 b	0.47 ± 0.13
TR10 (Ca:Mg 20:1)	0.023 ± 0.006 cd	0.023 ± 0.004 bcd	53.92 ± 14.66	21.66 ± 7.37	75.58 ± 21.09 b	0.4 ± 0.11
*p*-value	<0.001 ***	<0.001 ***	NS (0.103)	NS (0.112)	0.042 *	NS (0.101)

“NS” denotes no significant difference at a significant level of 95%; “*” denotes a significant difference at a significant level of 95%; “***” denotes a significant difference at a significant level of 99.9%.

**Table 4:** Leaf nutrient levels before and after application of Ca and Mg in the soil mixture. Mean comparison between before and after application was tested by *t*-test while mean comparison among treatment was tested by one-way ANOVA and Tukey’s post hoc test. The different lowercase letters indicate the mean difference at a significant level of 95% (*p*-value < 0.05). * represents a significant level of *p*-value < 0.05, ** a significant level of *p*-value < 0.01, and *** a significant level of *p*-value < 0.001.

Nutrients/Treatment	N (%)			P (%)			K (%)			Ca (%)			Mg (%)		
	Before	After	*t*-Test	Before	After	*t*-Test	Before	After	*t*-Test	Before	After	*t*-Test	Before	After	*t*-Test
TR1	0.69 ± 0.01 ab	0.57 ± 0.04 a	<0.001 ***	0.09 ± 0.01 a	0.05 ± 0.01 c	0.001 ***	1.43 ± 0.12 a	1.07 ± 0.04 abc	0.001 ***	0.48 ± 0.09 b	0.51 ± 0.05 abc	0.465	0.30 ± 0.03 ab	0.22 ± 0.01 cd	0.001 ***
TR2	0.66 ± 0.02 abcd	0.51 ± 0.03 bcd	<0.001 ***	0.09 ± 0 a	0.05 ± 0.01 c	<0.001 ***	1.31 ± 0.02 abcd	1.12 ± 0.12 ab	0.031 *	0.42 ± 0.02 bc	0.53 ± 0.04 abc	0.001 ***	0.30 ± 0.01 ab	0.24 ± 0.01 bcd	0.001 ***
TR3	0.69 ± 0.05 a	0.54 ± 0.04 abc	<0.001 ***	0.07 ± 0.02 ab	0.06 ± 0.01 bc	0.825	1.37 ± 0.10 ab	1.13 ± 0.13 ab	0.020 *	0.46 ± 0.07 bc	0.63 ± 0.06 a	0.003 **	0.26 ± 0.02 b	0.23 ± 0.01 bcd	0.006 **
TR4	0.64 ± 0.06 bcd	0.48 ± 0.01 d	<0.001 ***	0.04 ± 0.02 b	0.05 ± 0.01 c	0.740	1.40 ± 0.13 a	1.18 ± 0.08 a	0.007 **	0.46 ± 0.01 bc	0.55 ± 0.08 ab	0.077	0.26 ± 0.01 b	0.23 ± 0.01 bcd	0.001 ***
TR5	0.63 ± 0.04 cde	0.50 ± 0.02 bcd	<0.001 ***	0.05 ± 0.01 b	0.07 ± 0.02 abc	0.067	1.16 ± 0.06 cd	1.14 ± 0.10 ab	0.712	0.49 ± 0.04 ab	0.48 ± 0.05 bcd	0.776	0.28 ± 0.02 ab	0.20 ± 0.02 d	0.001 ***
TR6	0.61 ± 0.02 e	0.52 ± 0.02 abcd	<0.001 ***	0.04 ± 0.01 b	0.08 ± 0.02 ab	0.015 *	1.33 ± 0.10 abc	1.00 ± 0.05 bc	0.001 ***	0.34 ± 0.04 cd	0.42 ± 0.03 cd	0.008 **	0.28 ± 0.00 ab	0.26 ± 0.02 abc	0.112
TR7	0.64 ± 0.02 abcde	0.52 ± 0.03 abcd	<0.001 ***	0.04 ± 0.01 b	0.08 ± 0.02 ab	0.018 *	1.22 ± 0.19 bcd	0.96 ± 0.07 cd	0.007 **	0.23 ± 0.06 d	0.38 ± 0.05 d	0.002 **	0.28 ± 0.00 ab	0.26 ± 0.03 abc	0.316
TR8	0.61 ± 0.01 de	0.52 ± 0.04 abcd	0.005 ***	0.05 ± 0.01 b	0.08 ± 0.01 ab	<0.001 ***	1.15 ± 0.05 d	0.82 ± 0.05 de	0.001 ***	0.35 ± 0.01 bcd	0.36 ± 0.05 d	0.900	0.27 b ± 0.02	0.29 ± 0.05 a	0.544
TR9	0.67 ± 0.01 abc	0.49 ± 0.04 cd	<0.001 ***	0.04 b ± 0.01	0.08 ± 0.01 ab	<0.001 ***	1.32 ± 0.08 abcd	0.80 ± 0.12 e	0.001 ***	0.62 ± 0.01 a	0.48 ± 0.16 bcd	0.174	0.33 ± 0.02 a	0.24 ± 0.02 bcd	0.001 ***
TR10	0.66 ± 0.02 abcde	0.55 ± 0.05 ab	0.006 ***	0.05 ± 0.02 b	0.09 ± 0.03 a	0.035 *	1.42 ± 0.09 a	1.07 ± 0.09 abc	0.001 ***	0.37 ± 0.01 bc	0.55 ± 0.09 ab	0.007 **	0.28 ± 0.02 ab	0.27 ± 0.02 ab	0.356
*p*-value	0.020 *	<0.001 ***		<0.001 ***	<0.001 ***		0.021 *	<0.001 ***		<0.001 ***	<0.001 ***		<0.001 ***	<0.001 ***	

Green indicates statistical significance, with “a” denoting a very high quantity, while red represents *p*-values that indicate statistical differences. The blue color defines the statistical significance in mean difference among treatments.

**Table 5:** NMDS analysis indicating the influence of each soil parameter on kratom growth and mitragynine production in terms of *p*-values. Boldface and asterisks (*) denote statistically significant variables (*p*-value < 0.05) affecting plant development and alkaloid concentration.

Soil Chemical Parameters	*p*-Value
Soil pH	0.45
Soil N [%]	0.59
Soil P [mg/kg]	0.61
Soil K [mg/kg]	0.62
Soil Ca [mg/kg]	0.46
**Soil Mg [mg/kg]**	0.003 *
**Ca:Mg ratio**	0.04 *

**Table 6:** Preliminary field-testing results for soil parameters after a month following application of Ca and Mg in a ratio of 10:1.

Parameter	Control	Treatment
Soil pH	5.5–6.6	5.0–5.9
Exchangeable Ca (mg kg^−1^)	680–927	2200–7820
Exchangeable Mg (mg kg^−1^)	Not specified	Slightly lower in some treated soils
Ca:Mg Ratio	0.71–0.87	2.3–8.6
Soil Organic Matter	Baseline	Comparable to control
Total Carbon	Baseline	Comparable to control
Total Nitrogen	Baseline	Comparable to control

**Table 7:** Preliminary field-testing results for growth parameters and mitragynine content after one month following application of Ca and Mg in a ratio of 10:1.

Parameter	Control	Treatment	*p*-Value
Relative Height Growth Rate (m)	0.016 ± 0.013	0.052 ± 0.016	<0.05
Relative Diameter Growth Rate (cm)	0.018 ± 0.013	0.151 ± 0.080	<0.05
Foliar Mitragynine Content (% dry weight)	0.68 ± 0.12	0.82 ± 0.13	0.040

**Table 8:** Treatment groups assigned to various levels of calcium and magnesium supplementation to determine their influence on kratom seedlings. Each group consisted of 15 plants receiving different calcium (Ca) and magnesium (Mg) concentrations or Ca:Mg ratios, with the control group receiving no additional nutrients.

Treatment Group	Treatment Description	Ca (ppm)	Mg (ppm)	Remarks
TR1	Control (no addition)	0	0	No Ca or Mg added.
TR2	Ca Only—Medium	1000	0	Based on Kasetsart University [8]
TR3	Ca Only—High	2000	0	Based on Prince of Songkla University [12]
TR4	Ca Only—Very High	4000	0	High Ca concentration
TR5	Mg Only—Low	0	50	Low Mg levels
TR6	Mg Only—Optimal	0	175	Optimal Mg levels
TR7	Mg Only—Very High	0	400	Very high Mg levels [78]
TR8	Ca:Mg ratio 2:1	1000	500	[78]
TR9	Ca:Mg ratio 10:1	2000	200	[78]
TR10	Ca:Mg ratio 20:1	2000	100	[78]

## Data Availability

The original contributions presented in this study are included in the article. Further inquiries can be directed to the corresponding author.
